# How belief in a just world shapes academic engagement among Chinese college art majors: A cross-level moderated mediation model

**DOI:** 10.1371/journal.pone.0317583

**Published:** 2025-01-22

**Authors:** Jia Li, Junqing Bai, Lixia Ouyang, He Lin

**Affiliations:** 1 School of Music and Dance, Shanxi Vocational University of Engineering and Technology, Jinzhong, China; 2 College of Textile and Fashion, Hunan Institute of Engineering, Xiangtan, China; 3 Sport Art College, Guangzhou Sport University, Guangzhou, China; Pingdingshan University, CHINA

## Abstract

The belief in a just world (BJW) is perceived as an individual trait that aids in coping with challenges. This study employed Mplus8.0 and HLM6.08 to analyze 346 questionnaire responses, leading to the following conclusions: (1) BJW shows a positive correlation with academic engagement among college art majors; (2) Academic resilience mediates the relationship between BJW and academic engagement for college art majors; (3) The teacher-student relationship (TSR) exhibits a positive correlation with academic engagement among college art majors; (4) TSR functions as a moderator in the relationship between BJW and academic engagement among college art majors. These findings provide valuable insights for enhancing learning efficiency and talent development in art schools, thereby contributing to the overall quality of education for art students.

## Introduction

The belief in a just world (BJW) was proposed by Lerner and Miller [1]. Its core concept is that individuals believe the world is fair and people get what they deserve. Research on BJW mainly focuses on its positive effects on mental and physical development. BJW is seen as a personal trait with adaptive functions and serves as the foundation for coping strategies in difficulties and threats [2]. For example, there is evidence suggesting that BJW is associated with positive emotions, well-being, and psychological health [3–5], as well as lower levels of anxiety, depression, and distress [6–8]. Numerous studies have investigated BJW [9]. However, limited research has been done on its development in adolescents, particularly in non-Western contexts. Scholars have criticized existing theories and findings based on the "WEIRD" population (Western, Educated, Industrialized, Rich, and Democratic), questioning their bias. This bias applies to the study of BJW too. Shek et al. [10] highlighted the difficulty in generalizing findings from Western contexts due to unique cultural, political, economic, and social factors. Therefore, studying non-WEIRD samples is crucial, especially among Chinese individuals, representing the world’s largest population [11].

Academic engagement refers to a sustained and positive emotional state during academic pursuits, represented by vigor, dedication, and absorption [12]. Vigor shows resilience, the ability to persist and energetically engage in learning despite challenges. Dedication reflects strong enthusiasm for learning, experiencing meaning and value in the process. Absorption indicates a high level of focus, learning wholeheartedly and without distraction [13]. There is growing evidence that student engagement is crucial for successful learning and teaching [14]. Higher engagement is linked to positive learning outcomes [15, 16]. However, enhancing students’ engagement remains a persistent issue for educators and researchers in education. The current discussion among scholars on academic engagement mainly examines its relationship with social and environmental factors from an ecological systems theory perspective. For example, research has found that teacher support and classroom atmosphere can positively influence student academic engagement [17], and that higher levels of academic engagement are associated with higher academic achievement and performance, as well as lower academic burnout and dropout rates [18, 19]. Relevant studies provide useful references for inspiring students’ learning enthusiasm and improving academic achievement, but there are also several shortcomings. First, discussions on academic engagement mainly focus on the population of middle school students [20, 21], with less attention given to college students. Especially for art college students, who generally exhibit characteristics such as poor cultural foundation, insufficient learning motivation, and lack of academic engagement [22]. Therefore, further exploration is needed to determine whether research conclusions based on the middle school student population are applicable to art college students [23]. Second, college students are at a critical period for the formation and stability of their BJW [20], and this belief has an important impact on the physical and mental development of college students, such as problem behavior, positive emotions, and psychological well-being, and it also predicts college students’ academic performance [21]. However, there is relatively little research on using BJW as a factor influencing academic engagement at this stage.

Academic resilience, the ability to improve academic performance after setbacks, is defined as a student’s capacity to rebound from adverse events [24]. The connection between resilience and academic performance has produced inconsistent findings. Tempski et al. [25] found a significant association between high resilience and a positive perception of learning environments among students. On the other hand, Elizondo-Omana et al. [26] revealed no correlation between resilience score and academic performance, while other studies suggested a weak relationship [27, 28]. What is the relationship between academic resilience and academic achievement? This necessitates further research and exploration.

Teacher-student relationships (TSRs) are central to students’ schooling experience [29]. Noddings [30] suggests that strong teacher-student connections enable responsive and sensitive instruction. Research indicates that these relationships develop through ongoing interactions and the nuanced meanings teachers convey to students [31]. Moreover, the accuracy of students in understanding their teachers’ perspectives and their perceptions of teachers’ long-term behavior are critical in shaping these relationships [31, 32]. Furthermore, patterns of teacher-student interactions are influenced by both partners’ actions and reactions, which, in turn, are shaped by their perceptions and interpretations of each other’s behavior [33]. Teachers’ expectations and evaluations of student behavior can impact student development and the quality of these relationships [34, 35]. Positive TSRs predict enhanced student engagement and academic achievement [36, 37]. Therefore, TSRs may play a significant role in the relationship between BJW and academic engagement. However, existing research primarily analyzes TSRs as individual-level variables [38–40]. In practical research, it is crucial to rigorously assess differences in variables at different levels and the suitability of a general regression model. Therefore, when exploring the role of TSRs in the relationship between BJW and academic engagement, relying solely on subjective judgment for single-level analysis methods is insufficient.

Based on the aforementioned analysis, this study employs a multilevel linear model to empirically analyze the mechanisms of BJW, academic resilience, TSRs, and students’ academic engagement. The findings of this study can provide references for improving learning efficiency among college students in art schools and enhancing the quality of talent cultivation in art schools.

## Theoretical review and research hypotheses

### Individual level

Academic engagement refers to a positive emotional state displayed by individuals during learning. It includes the time, energy, and degree students invest in cognition, emotion, and behavior [41]. Cognitive engagement refers to learners’ intrinsic motivation and use of deep learning methods. Emotional engagement refers to individuals interested in learning and gaining satisfaction. It also includes teacher-student and student-student relationships. Behavioral engagement refers to learners’ participation in learning activities [42, 43]. BJW promotes learning motivation, attention to academic goals, effective time management, and increased learning investment, thereby improving academic achievement. Previous studies suggest that academic engagement may be influenced by BJW. Lerner et al argue that this belief promotes the pursuit of long-term goals [44] and increases confidence in the future [45]. The stronger the belief, the more efficiently individuals manage their time, increasing study time and reducing leisure time [46]. On the contrary, perceived injustice reduces motivation and concern for academic progress [47]. Therefore, we propose the following hypothesis.

Hypothesis 1: BJW shows a positive correlation with academic engagement among college art majors.

The Self-Efficacy Theory suggests that an individual’s belief in a just world can influence their academic resilience by affecting their confidence and evaluation of their abilities in academics [48]. If individuals can accept the existence of academic challenges and commit to perseverance in their studies, they are more likely to overcome difficulties and enhance their academic resilience [49, 50]. Additionally, Acceptance and Commitment Therapy (ACT) also emphasizes that an individual’s belief in a just world can influence their academic resilience by influencing their acceptance of academic difficulties and their commitment to continuous learning [51, 52].

The Job Demand-Resource (JD-R) model, suggests that internal psychological resources play a crucial role in coping with work environments, reducing burnout, and enhancing work engagement [53]. Academic resilience is influenced by protective and vulnerable factors, both internal and external, that influence individuals’ ability to adapt to challenges and stressors [54]. According to Brewer et al. [55] and Davydov et al. [56], academic resilience is a positive psychological resource that helps individuals overcome challenging situations. It reflects learners’ capacity to handle academic difficulties [55]. In college, students face both positive and negative experiences, requiring adaptation to different levels of adversity [57]. Studies have shown positive links between academic resilience and performance [58], and negative associations with stress, anxiety [57], and school failure [59]. Cheng and Huang [60] found that higher psychological resilience is associated with higher academic resilience, enabling individuals to cope effectively with setbacks and achieve better academic performance. Therefore, psychological resilience significantly impacts college students’ academic engagement. Based on this, the following hypotheses are proposed:

Hypothesis 2: Academic resilience mediates the relationship between BJW and academic engagement for college art majors.

### Organizational level

A robust teacher-student bond can establish a secure environment that promotes active participation in classroom activities. Pitzer & Skinner [61] assert that students’ perceptions of their interactions with teachers and their self-perceptions are shaped by the fulfillment of their needs through contextual support, with these reciprocal effects significantly impacting student engagement. Furthermore, factors such as alleviating negative emotions [62], teacher feedback [63], and teacher behavior [64] have been identified as influencing the correlation between TSRs and student engagement. Positive TSRs, particularly those related to emotions [65, 66], augment the influence of external factors on student engagement. Additionally, teachers’ evaluation and decision-making regarding student performance in the classroom [67, 68] directly impact students’ educational trajectories. Harmonious TSRs, which encompass emotional, instrumental, and informational support [69, 70], foster feelings of pleasure, respect, and trust [71], which are vital components of school social capital. These relationships also enhance students’ motivational beliefs [72, 73] and contribute to classroom engagement [72, 74, 75], autonomy in learning [71], and positive academic emotions such as increased enjoyment [69, 76] and reduced anxiety [77]. Consequently, positive TSRs play a critical role in students’ academic success [69, 73, 74, 78]. Therefore, we propose the following hypothesis.

Hypothesis 3: TSR exhibits a positive correlation with academic engagement among college art majors.

Social cognitive theory is widely used to understand human behavioral change, involving the interplay of individual factors, behavioral changes, and environmental influences [79–81]. Human behavior is motivated and regulated by both internal personal factors and external environmental factors [79, 80, 82]. The TSR is particularly significant among these external factors [83], and positive relationships can lead to beneficial changes in students’ behaviors [84, 85]. BJW serves a crucial trust function, enabling individuals to trust others and have confidence in a just fate [86]. College students with BJW believe that their efforts will yield positive academic outcomes. Positive TSRs contribute to students feeling supported and cared for [87] and developing positive attitudes [88, 89]. Teachers play a vital role in providing academic guidance, emotional support, and encouragement, especially for students facing learning challenges, which enhances their social capital [90, 91]. Students can seek academic and emotional support from teachers when facing challenges, leading to improved learning outcomes [90]. In addition, positive TSRs, characterized by familiarity, high expectations, attentiveness, and increased interaction, enhance classroom participation, learning effectiveness, and students’ learning initiative [88]. In other words, having a positive TSR increases the likelihood of teachers providing academic and emotional support, enhances students’ self-efficacy [79, 92], strengthens the belief that high academic engagement leads to rich academic outcomes, and reinforces the relationship between BJW and college students’ academic engagement. Conversely, tense TSRs lead to negative emotions, reduced academic and emotional support, and shake the belief that high academic engagement leads to rich academic outcomes, weakening students’ self-efficacy and dampening the relationship between BJW and college students’ academic engagement. Therefore, we propose the following hypothesis.

Hypothesis 4: TSR functions as a moderator in the relationship between BJW and academic engagement among college art majors.

**Fig 1:**
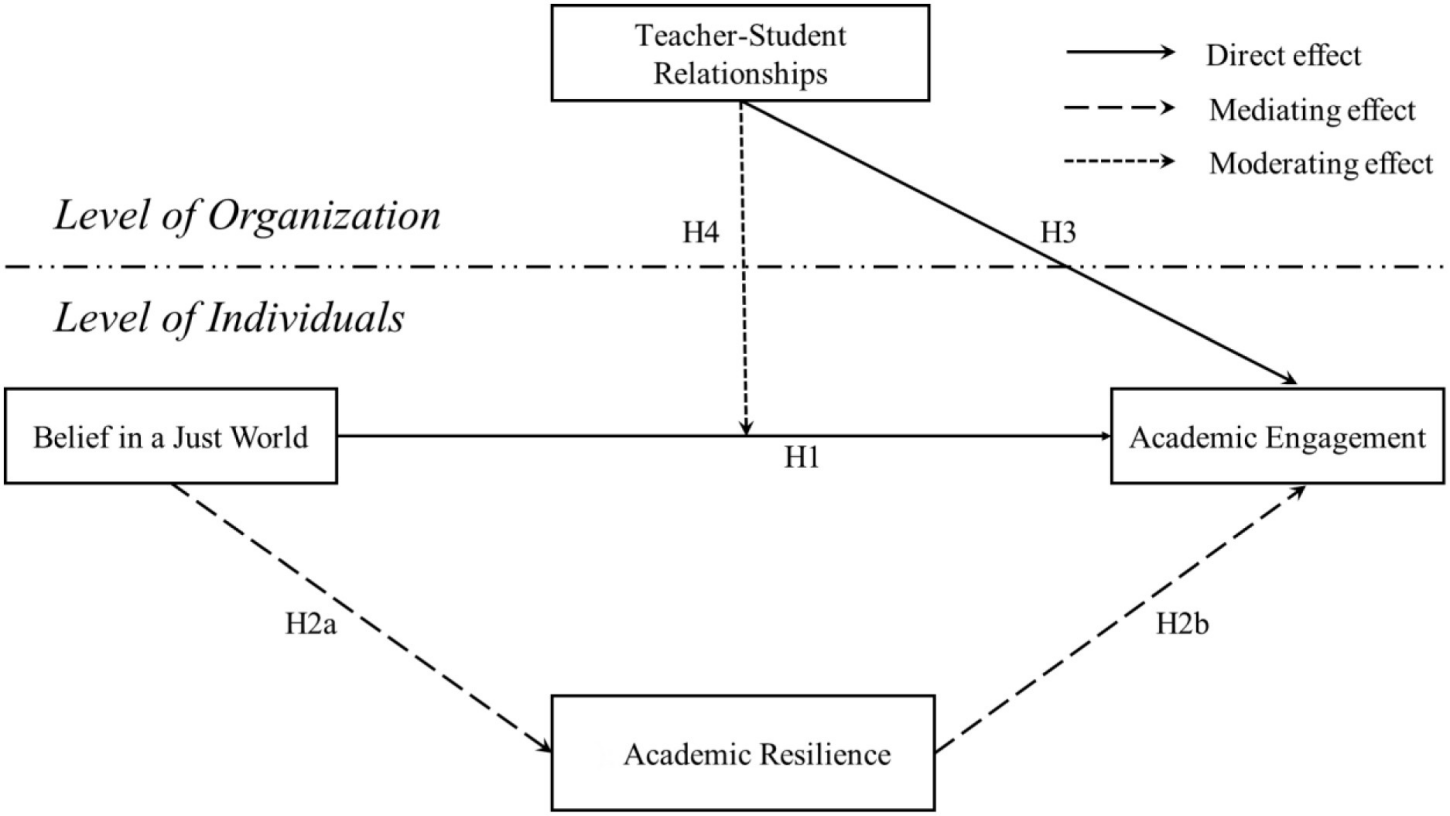
Hypothetical model is presented here for illustration.

The hypothetical model of the paper is shown in Fig 1.

## Methods

### Data collection

Data collection was conducted through a questionnaire survey from June 12, 2023, to August 24, 2023, and consisted of two parts. The first part elucidated the study’s purpose and significance, assuring respondents that their data would be used solely for scientific research and not for commercial purposes. It also clarified the exceptions under which participants could refuse to complete the questionnaire: first, if they had doubts about the study’s purpose and significance; second, if they disagreed with the use of the questionnaire data for this research; and third, according to the Chinese education system, the enrollment age for first grade is 7 years old, followed by 6 years of primary school and 6 years of secondary school. Therefore, university students would all be over 18 years of age (the age of majority). However, to avoid any exceptions, we explicitly stated that participants under the age of 18 could refuse to complete the questionnaire or do so only with written consent from their parents or guardians. Additionally, at the end of this section, we included an informed consent clause to ensure that our research obtained informed consent from each participant. The second part encompassed various variables. The measurement of relevant variables was conducted using well-established and widely recognized scales. We invited professional translators to translate these scales into Chinese and refine them to ensure they align with the language habits and expression styles of Chinese respondents. To minimize respondents’ guessing of item answers, we randomized the order of the items.

We selected 20 colleges and universities offering art programs, with 9 situated in the eastern region of China, 7 in the central region, and 4 in the western region. Out of these institutions, we randomly selected 60 art teachers. Each teacher was then asked to randomly invite 6–8 art students they taught to complete the questionnaire. We used Wenjuanxing, a highly popular online survey tool in China, for data collection. The data collection was conducted in a single session, with each questionnaire requiring no more than 10 minutes to complete. Participants who completed the questionnaire received a small gift valued at $2. A total of 440 questionnaires were distributed, and 402 questionnaires were eventually collected. We subsequently followed the following criteria to exclude invalid questionnaires. First, incomplete questionnaires were removed. Second, questionnaires with logical errors were excluded. Third, questionnaires collected from fewer than 5 students taught by the same teacher were eliminated. After removing the invalid questionnaires, 346 questionnaires from 52 art teachers were retained. Each teacher had at least 5 valid questionnaires. This sample size meets the recommended standards of Cora & Hox [93]. Among the 346 questionnaires, there were 231 female students and 115 male students; the average age was 20.3 years with a standard deviation (SD) of 1.6 years. Among the respondents, 219 were from undergraduate institutions and 127 were from vocational colleges.

### Measure

BJW was assessed using an adapted scale from Dalbert’s [94] research, consisting of 7 items for measuring BJW-self (e.g., "Overall, events in my life are just") and 6 items for assessing BJW-other (e.g., "I think basically the world is a just place").

For the measurement of academic resilience, refer to the Academic Resilience Scale (ARS-30) compiled by Cassidy [24], which is divided into Perseverance (e.g., "I would work harder"), Reflecting and adaptive help-seeking (e.g., "I would give myself encouragement"), and Negative affect and emotional response (e.g., "I would probably get depressed"), with a total of 30 items.

Since students above grade 3 can accurately report their perception of TSRs [95], and previous research primarily employed student self-perception [69], this study utilized students’ self-reported TSRs. This research used adapted items from PISA, which have been previously validated for reliability and validity [78]. There are five items, for example, "I get on well with teachers."

Academic engagement was evaluated with the reduced version of the Utrecht Work Engagement Scale–UWES—version for students [96]. The reduced version of this measure consists of 9 items that measure the factors Vigor (e.g., “My activities as a student make me feel full of energy”), Dedication (e.g., “My studies inspire new things for me”), and Absorption (e.g., “I ‘let myself go’ when I perform my activities as a student”).

We utilized a 7-point Likert scale to assess all items, with 1 representing strong disagreement and 7 indicating strong agreement.

### Tools

In this study, Mplus8.0 and HLM6.08 were used for data analysis.

## Results

### Reliability and validity

Table 1 displays the variables’ reliability and validity. All measurement items have factor loads exceeding 0.685. The variables’ CR ranges from 0.798 to 0.854, and the AVE is above 0.540, demonstrating favorable reliability and convergent validity. Table 2 exhibits the correlation coefficients between variables, indicating sound discriminant validity.

**Table 1:** Results of confirmatory factor analysis and reliability and validity tests.

Dim	Item	Parameters of significant test			Item	Composite	Convergent
					Reliability	Reliability	Validity
		Estimate	SE	P-value	SMC	CR	AVE
BJW	BJW1	0.837	.022	***	0.701	0.798	0.665
	BJW2	0.793	.026	***	0.629		
AR	AR1	0.772	.021	***	0.596	0.827	0.615
	AR2	0.815	.017	***	0.664		
	AR3	0.764	.019	***	0.584		
TSR	TSR1	0.753	.023	***	0.567	0.854	0.540
	TSR2	0.797	.025	***	0.635		
	TSR3	0.739	.024	***	0.546		
	TSR4	0.685	.019	***	0.469		
	TSR5	0.693	.023	***	0.480		
AE	AE1	0.764	.016	***	0.584	0.811	0.589
	AE2	0.735	.019	***	0.540		
	AE3	0.802	.017	***	0.643		

Note 1: BJW = belief in a just world; AR = academic resilience; TSR = teacher-student relationship; AE = academic engagement. The same below. Note 2: ***P < 0.001; **P < 0.01; *P < 0.05, the same below.

**Table 2:** Correlation coefficients and the square root of the average variance extracted.

Dim	Mean	SD	Convergent Validity	Discriminant Validity			
			AVE	BJW	AR	TSR	AE
BJW	4.749	0.611	0.665	**0.815**			
AR	4.954	0.807	0.615	0.492	**0.784**		
TSR	4.417	0.742	0.540	0.249	0.177	**0.735**	
AE	4.573	0.778	0.589	0.196	0.355	0.531	**0.767**

The bold diagonal font is the square root value of the average variance extracted (AVE), and the lower triangle shows Pearson’s correlation coefficients.

### Basic characteristic test

TSR in this study belongs to the shared construct. In practice, *r*_*wg*_ is considered acceptable if it is greater than 0.70 [97]. In this study, the average *r*_*wg*_ of TSR is 0.807, which meet relevant requirements.

### Hypothesis testing

#### Null model

The analysis revealed within-group component (σ^2^) as 0.471, between-group component (τ_00_) as 0.108, and ICC1 as 0.187. According to Cohen [98], these values indicate a high correlation, suggesting significant group differences, rendering a general regression model unsuitable.

Level 1: ${AE}_{ij}=\beta_{0j}+r_{ij}$
Level 2: $\beta_{0j}=\gamma_{00}+u_{0j}$
Mixed Model: ${AE}_{ij}=\gamma_{oo}+u_{oj}+r_{ij}$

#### Random coefficients regression model

The correlation analysis model is shown below. In this model, both BJW and academic resilience were group-centered. Table 3 displays the results, supporting H1.

Level 1: ${AE}_{ij}=\beta_{0j}+\beta_{1j}\left({BJW}_{ij}-{\bar{BJW}}_{.j}\right)+\beta_{2j}\left({AR}_{ij}-{\bar{AR}}_{.j}\right)+r_{ij}$
Level 2: $\beta_{0j}=\gamma_{00}+u_{0j};\;\beta_{1j}=\gamma_{10}+u_{1j};\;\beta_{2j}=\gamma_{20}+u_{2j}$;
Mixed Model:
$$\begin{array}{l} AE_{ij}=\gamma_{00}+u_{0j}+\gamma_{10}\left(BJW_{ij}-{\bar{BJW}}_{.j}\right)+u_{1j}\left(BJW_{ij}-{\bar{BJW}}_{.j}\right)+\gamma_{20}\left(AR_{ij}-{\bar{AR}}_{.j}\right)\\ \;+u_{2j}\left(AR_{ij}-{\bar{AR}}_{.j}\right)+r_{ij} \end{array}$$

**Table 3:** Results of random coefficients regression model.

	Effect	SE	P-value	Hypothesis(Y/N)
γ_00_	2.717	0.283	***	
γ_10_	.157	0.034	***	H1 (Y)
γ_20_	.385	0.033	***	

Table 4 displays the total effect of BJW on academic engagement as 0.257 (P < .001), comprising a direct effect of 0.155 (P < .001) and an indirect effect of 0.101 (P < .001), supporting H2.

**Table 4:** Test of mediating effect.

	Estimate	SE	P-Value	Hypothesis (Y/N)
Total	0.257	0.044	***	
Dir	0.155	0.038	***	
Ind: BJW->AR->AE	0.101	0.029	***	H2 (Y)

### Intercepts as outcomes model

#### Intercepts as outcomes model

For a deeper analysis of the organizational level TSR’s main effects, intercepts were examined as an outcomes model. The corresponding analysis model is presented here, with the results displayed in Table 5, supporting H3.

Level 1: ${AE}_{ij}=\beta_{0j}+r_{ij}$
Level 2: $\beta_{0j}=\gamma_{00}+\gamma_{01}\left({TSR}_{j}-{\bar{TSR}}_{.}\right)+u_{0j}$
Mixed Model: ${AE}_{ij}=\gamma_{00}+\gamma_{01}\left({TSR}_{j}-{\bar{TSR}}_{.}\right)+u_{0j}+r_{ij}$

**Table 5:** Results of intercepts as outcomes model.

	Effect	SE	P-value	Hypothesis (Y/N)
γ_00_	2.611	0.347	***	
γ_01_	0.409	0.047	***	H3(Y)

#### Slope as outcomes model

**Fig 2:**
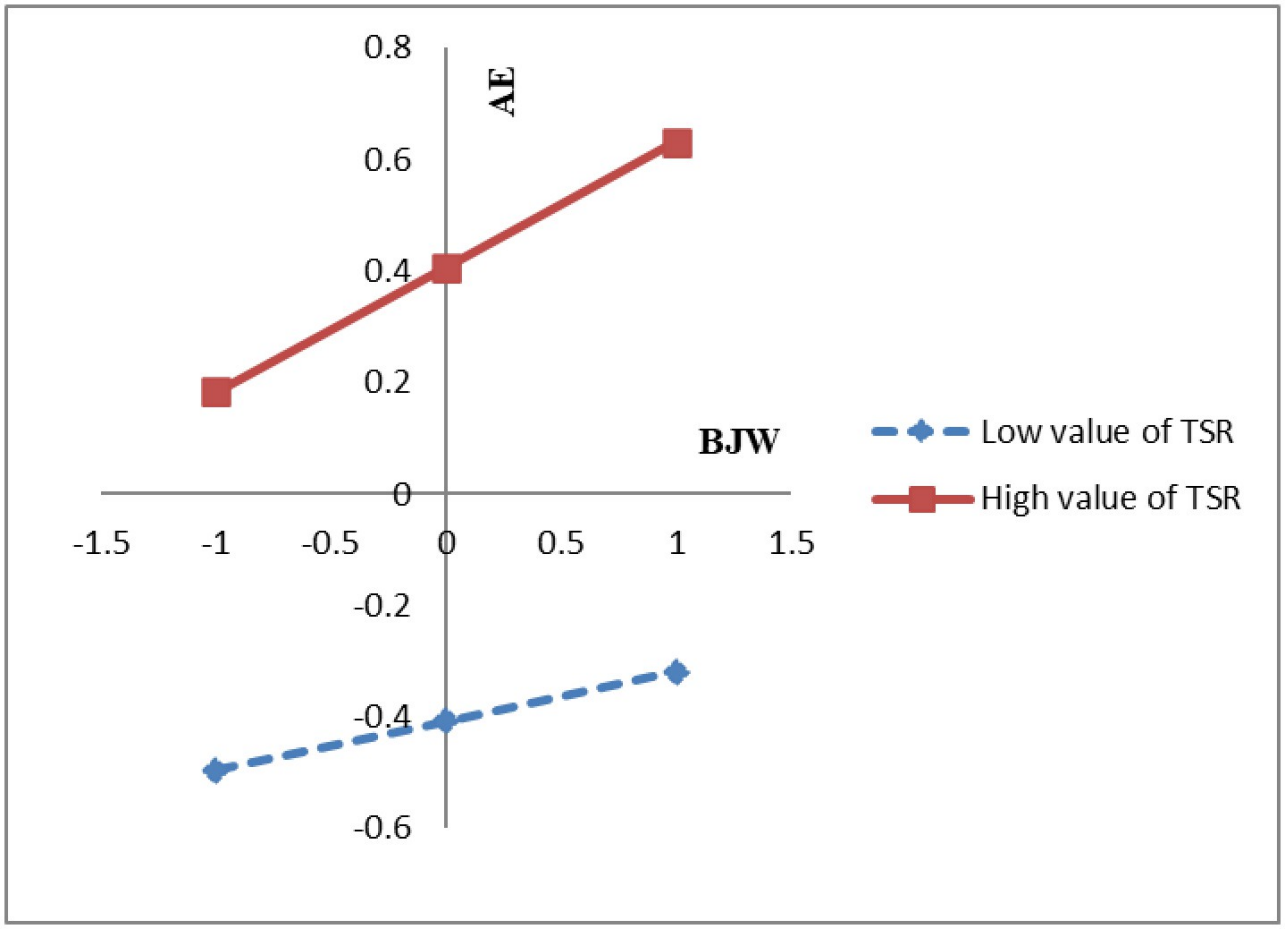
The moderating effect of TSR is presented here for illustration.

The correlation analysis model is displayed below, with the results presented in Table 6, supporting H4. Fig 2 shows the moderating effect of TSR. By comparison, the gradient of the slope for a high TSR was larger than that of a low TSR. This suggests that a stronger TSR corresponds to a greater influence on the BJW-academic engagement relationship.

Level 1: ${AE}_{ij}=\beta_{0j}+\beta_{1j}\left({BJW}_{ij}-{\bar{BJW}}_{.j}\right)+\beta_{2j}\left({AR}_{ij}-{\bar{AR}}_{.j}\right)+r_{ij}$
Level 2: $\beta_{0j}=\gamma_{00}+\gamma_{01}\left({TSR}_{j}-{\bar{TSR}}_{.}\right)+u_{0j}$;
  $\beta_{1j}=\gamma_{10}+\gamma_{11}\left({TSR}_{j}-{\bar{TSR}}_{.}\right)+u_{1j}$;
  $\beta_{2j}=\gamma_{20}+u_{2j}$;
Mixed Model:
$$\begin{array}{l} AE_{ij}=\gamma_{00}+\gamma_{01}\left(TSR_{j}-{\bar{TSR}}_{.}\right)+\gamma_{10}\left(BJW_{ij}-{\bar{BJW}}_{.j}\right)+\gamma_{11}\left(TSR_{j}-{\bar{TSR}}_{.}\right)\left(BJW_{ij}-{\bar{BJW}}_{.j}\right)\\ \;+\gamma_{20}\left(AR_{ij}-{\bar{AR}}_{.j}\right)+u_{0j}+u_{1j}\left(BJW_{ij}-{\bar{BJW}}_{.j}\right)+u_{2j}\left(AR_{ij}-{\bar{AR}}_{.j}\right)+r_{ij} \end{array}$$

**Table 6:** Results of slope as outcomes model.

	Effect	SE	P-value	Hypothesis(Y/N)
γ_00_	0.872	0.122	***	
γ_01_	0.407	0.045	***	
γ_10_	0.156	0.032	***	
γ_11_	0.068	0.025	**	H4(Y)
γ_20_	0.383	0.031	***	

## Discussion

### Theoretical contribution

Drawing on China’s social and cultural context, this study extends the investigation into the relationship between BJW and academic engagement by focusing on art students, thereby expanding the applicability of BJW and academic engagement theories.

Secondly, this study confirms a significant correlation between academic resilience and academic engagement. This conclusion supports the views of Tempski et al. [25], but it differs from the perspectives of other scholars [26–28].

Finally, this study found that TSR, as a shared construct, differences in variables at different levels cannot be subjectively ignored, and its role cannot be directly explored with a simple regression model [38–40]. Based on this, it is necessary to further use the HLM model for analysis and discussion.

### Practical implications

First, actively strengthen college students’ BJW: Firstly, provide a wide range of social education to promote intercultural understanding and respect. This can be achieved through lectures, colloquiums, or seminars aiming to educate students about concepts of justice and equality in different cultural contexts. Secondly, encourage participation in social practice by organizing students to engage in public welfare activities or volunteer services. This allows them to personally experience social injustice and inequality, stimulating their awareness of social justice. Thirdly, in-depth classroom education should be utilized. Within classroom teaching, teachers can guide students to contemplate the importance of justice and equality, stimulating students’ thinking and discussions through case analysis and other approaches.

Secondly, efforts should be made to enhance the academic resilience of college students: Firstly, provide mental health support. Colleges and universities can strengthen mental health education, offer psychological counseling and counseling services, and help students build a positive attitude to cope with challenges. Secondly, cultivate problem-solving abilities. The curriculum and teaching methods of colleges and universities should encourage students to flexibly cope with difficulties and challenges and cultivate their ability to overcome academic hardships. Thirdly, encourage the recognition of learning outcomes. Colleges and universities can establish a reward mechanism for academic achievement to encourage students to work hard and improve their academic performance.

Finally, strive to establish a good TSR: Firstly, create an open communication atmosphere where teachers listen to students’ ideas and suggestions, respect their individual differences, and encourage students to express their own views. Secondly, provide support and guidance as teachers should care about the growth and development of students and provide academic and life support and guidance. Lastly, establish cooperative relations. Encourage cooperation and interaction between teachers and students to enhance understanding and trust, facilitating mutual growth and progress.

### Limitations and future directions

Firstly, although the relevant variables in this study demonstrate high reliability and validity, the use of cross-sectional data introduces certain limitations when discussing causal relationships. These limitations stem from the lack of temporal information, issues of endogeneity, simultaneity bias, and the inability to control for time-varying factors. Therefore, to strengthen the research, it is essential to expand data sources and incorporate longitudinal data, as this will be critical for accurately inferring causality between variables.

Secondly, this study explores the internal mechanisms underlying the relationship between BJW and academic engagement. Future research should consider integrating additional factors, such as family background, social and cultural background, professional characteristics, and other relevant variables, to comprehensively evaluate the impact of BJW on college students’ academic engagement. Additionally, the generalizability of the findings to non-art students warrants further investigation.

Finally, while this study supports the notion of a significant correlation between academic resilience and academic engagement, it is important to note that it does not imply that the perspectives of other scholars [26–28] are incorrect. Instead, the focus should be on further investigating the reasons behind the differing conclusions in subsequent research.

## Conclusions

This study explores the relationship between Basic Job Worth and academic engagement among Chinese college art majors and reaches the following conclusions: (1) BJW shows a positive correlation with academic engagement among college art majors; (2) Academic resilience mediates the relationship between BJW and academic engagement for college art majors; (3) The teacher-student relationship (TSR) exhibits a positive correlation with academic engagement among college art majors; (4) TSR functions as a moderator in the relationship between BJW and academic engagement among college art majors. These findings provide insights for improving learning efficiency and talent development in art schools, thereby contributing to the enhancement of education quality for art students.
