# Correction: How belief in a just world shapes academic engagement among Chinese college art majors: A cross-level moderated mediation model

**DOI:** 10.1371/journal.pone.0334071

**Published:** 2025-10-08

**Authors:** Jia Li, Junqing Bai, Lixia Ouyang, He Lin

There is an error in affiliation 1 for authors Jia Li and Junqing Bai. The correct affiliation 1 is: School of Music and Dance, Shanxi Vocational University of Engineering Science and Technology, Jinzhong, China.
